# Changes in the malignant female reproductive system tumors disease spectrum at the Beijing obstetrics and gynecology hospital in the past 60 years

**DOI:** 10.3389/fmed.2025.1643451

**Published:** 2025-10-15

**Authors:** Yue He, Sheng-Qian Wang, Chen Ji, Zhen-You Liu, Jia-Hui Wei, Ming Wang, Yu-Mei Wu, Yan Wang, Xiao-Hong Xu

**Affiliations:** ^1^Department of Gynecological Oncology, Beijing Obstetrics and Gynecology Hospital, Capital Medical University, Beijing Maternal and Child Health Care Hospital, Beijing, China; ^2^Department of Immunology, School of Basic Medical Sciences, Laboratory for Clinical Medicine, Capital Medical University, Beijing, China

**Keywords:** female reproductive system, cervical cancer, ovarian cancer, endometrial carcinoma, women’s health

## Abstract

**Objective:**

This study aimed to evaluate the epidemiological trends in the incidence, age distribution, and pathological types of malignant female reproductive system tumors over the past 60 years at the Beijing Obstetrics and Gynecology Hospital.

**Methods:**

The types of diseases and basic clinical information of 18,921 patients with malignant female reproductive system tumors admitted to our hospital between January 1960 and December 2019 were collected.

**Results:**

A total of 18,921 patients were diagnosed and treated in the last 60 years. Since the establishment of the Department of Gynecological Oncology at our hospital in 1970, the number of patients with gynecological tumors has doubled, and the number of tumor types has also increased, with the highest incidence of cervical cancer, followed by endometrial cancer, ovarian cancer, and trophoblastic tumors. The three most common tumor types were most prevalent among women aged over 36 years. The average age of patients was 49.43 ± 11.65 years for those with cervical cancer, 53.95 ± 9.45 years for endometrial cancer, and 43.04 ± 13.79 years for ovarian cancer. Over the last 20 years, the age of patients with cervical cancer has been decreasing, and the age of patients with endometrial or ovarian cancers has slowly increased. Squamous cell carcinoma (85.61%–100%) was the most prevalent cervical cancer subtype; while, adenocarcinoma (88%–100%) was the most common endometrial cancer subtype and epithelial carcinoma was the most common ovarian cancer subtype.

**Conclusion:**

The establishment of a gynecological oncology subspecialty correlated with improved diagnostic capabilities and a marked increase in the number of cases. The observed epidemiological shifts underscore the need for targeted screening programs, as well as preventive and control policies. Furthermore, the proportion, classification, and age distribution characteristics of malignant tumors in the female reproductive system changed over time. These findings provide a foundation for refining national cancer prevention policies.

## 1 Introduction

Cervical, endometrial, and ovarian cancers are the most prevalent malignancies of the female reproductive system. In 2020, an estimated 19.3 million new cancer cases and 10 million cancer-related deaths worldwide. The mortality rate in Asia (58.3%) exceeds the incidence rate (49.3%), accounting for 59.5% of the global population. In the same year, global data indicated that cervical cancer remained the fourth most common cancer among women, whereas endometrial and ovarian cancers ranked sixth and eighth, respectively (1). China shoulders a significant burden, with 17.51% of new global cases of female reproductive system malignancies. Cervical, endometrial, and ovarian cancers are ranked 6th, 9th, and 10th in global incidence, respectively (2). The total number of female reproductive system cancer-related deaths in China was 1,182,897, accounting for 26.71% of the global total. Among the top 10 most common malignant tumors in women, cervical cancer has the 7th highest mortality rate, accounting for 17.28% of the global total. Ovarian cancer ranks 9th, accounting for 18.10% of the cases worldwide (3). China has a high prevalence of cancer and malignant tumors of the female reproductive system seriously affect the health of Chinese women.

The Beijing Obstetrics and Gynecology Hospital was established in June 1959 and the Department of Gynecological Oncology was established in 1970 to focus on the diagnosis and treatment of malignant female reproductive system tumors. Our hospital is the sole specialized tertiary obstetrics and gynecology hospital in Beijing and serves as one of the key centers for the diagnosis and treatment of obstetric and gynecological diseases in Beijing. Our hospital manages one-third of all malignant female reproductive system tumors in Beijing, and 70% of the patients come from outside the city. The trends identified in this study are highly representative of the evolution of malignant female reproductive system tumors at our hospital over the past 60 years from 1960 to 2019. This underscores the importance of the development and advancement of gynecological oncology. We aimed to evaluate the impact of the establishment of the Department of Gynecological Oncology in 1970 on diagnostic capabilities and to identify trends in patient demographics and changes in disease patterns that could guide national prevention and control strategies. Our findings will provide a basis for refining existing screening guidelines, enabling accurate early detection and early warning for high-risk groups, enhancing the health status of Chinese women, and designing adjustments to prevention and control policies for tumors in the female reproductive system.

## 2 Materials and methods

### 2.1 Patients

This study included 18,921 patients with malignant tumors of the female reproductive system admitted to Beijing Obstetrics and Gynecology Hospital Affiliated with Capital Medical University between January 1960 and December 2019. Patients with two or more simultaneous gynecological cancers were included in groups based on the first cancer treated. If two or more pathological types were combined, the type with the highest proportion was recorded, and the criterion for each tumor was the lesion site of the primary tumor. The inclusion criteria were: (1) confirmed pathological diagnosis of a malignant tumor of the female reproductive system and (2) newly treated patients with complete case data. The exclusion criteria were: (1) incomplete medical history and (2) no pathological diagnosis or incomplete pathological diagnosis.

### 2.2 Research methods

Clinical data, including disease type, pathological type, and age of patients with malignant female reproductive system tumors at Beijing Obstetrics and Gynecology Hospital from January 1960 to December 2019 (data collection ended in December 2019).

The age distribution was based on China’s standardized age groups and the data were stratified by age groups (0–18, 19–35, 36–45, 46–55, 56–65, 66–75, ≥76 years) and decade of diagnosis. Groups were defined by age with children and teenagers being under 18 years old, young adults between 19 and 35 years old, middle-aged adults between 36 and 65 years old, and elderly people over 65 years old. Due to the large age span of middle adulthood, after 35 years of age, the participants were further divided into seven age groups: 0–18 years old, 19–35 years old, 36–45 years old, 46–55 years old, 56–65 years old, 66–75 years old, and ≥76 years old for age stratification.

Pathological specimens were reviewed by two senior pathologists.

Tumor staging data were not collected in this study. Staging records were incomplete in the early years owing to limited diagnostic techniques, and staging criteria (e.g., FIGO staging) were updated multiple times over 60 years, leading to non-standardized data across decades. Thus, the staging data were not included in the analysis. In future analyses, we will conduct in-depth research to collect relevant information for statistical analysis.

### 2.3 Statistical analysis

Statistical software (SPSS 21.0) was used to analyze the data. Qualitative data are expressed as rates or constituent ratios, and normally distributed measurement data are expressed as ($\bar{x}\;$± s). Quantitative data were analyzed using the Chi-square test.

## 3 Results

### 3.1 Analysis of malignant female reproductive system tumors at Beijing obstetrics and gynecology hospital over the last 60 years

Over the past 60 years, the Beijing Obstetrics and Gynecology Hospital has treated 18,921 patients with malignant tumors of the female reproductive system. The number of cases in each decade since 1960 was 73, 752, 2,207, 1,662, 4,165, and 10,062 for the 1960s, 1970s, 1980s, 1990s, 2000s, and 2010s, respectively. Since the creation of our Gynecological Oncology Department in 1970, the incidence of malignant tumors in the female reproductive system and the variety of tumor types have significantly increased. Cervical cancer has the highest incidence, followed by endometrial cancer, ovarian cancer, and trophoblastic tumors. Throughout the 60-year period, the types of gynecological cancers have evolved, with the incidence of cervical cancer gradually declining. In contrast, the incidences of endometrial and ovarian cancers increased annually (*p* < 0.05; Table 1).

**Table 1:** Changes in the number and types of malignant tumors of the female reproductive system (1960–2019), shows the number of major tumor types per decade.

Year	Disease												
	Cervical cancer	Endometrial cancer	Ovarian cancer	Trophoblastic tumor	Vulvar cancer	Uterine sarcoma	Follopian tube cancer	Vaginal cancer	Residual vaginal cancer	Peritoneal cancer	Total	Chi-square value	*p*-value
1960–1969	67 (91.78%)	6 (8.22%)	0	0	0	0	0	0	0	0	73 (0.39%)	1086.036	0.000
1970–1979	665 (88.4%)	77 (10.24%)	2 (0.27%)	0	2 (0.27%)	4 (0.55%)	0	0	2 (0.27%)	0	752 (3.97%)		
1980–1989	1,571 (71.18%)	331 (15.00%)	234 (10.60%)	14 (0.63%)	17 (0.77%)	15 (0.68%)	13 (0.58%)	11 (0.50%)	1 (0.04%)	0	2,207 (11.66%)		
1990–1999	649 (39.05%)	524 (31.53%)	325 (19.55%)	86 (5.17%)	28 (1.70%)	27 (1.62%)	14 (0.84%)	7 (0.42%)	2 (0.12%)	0	1,662 (8.78%)		
2000–2009	1872 (44.95%)	999 (23.99%)	837 (20.10%)	265 (6.36%)	65 (1.56%)	54 (1.30%)	40 (0.95%)	23 (0.55%)	5 (0.12%)	5 (0.12%)	4,165 (22.01%)		
2010–2019	5,370 (53.37%)	2,415 (24.00%)	1,521 (15.12%)	360 (3.58%)	140 (1.39%)	80 (0.80%)	103 (1.02%)	40 (0.40%)	19 (0.19%)	14 (0.13%)	10,062 (53.19%)		
Total	10,194 (53.88%)	4,352 (23.00%)	2,919 (15.43%)	725 (3.83%)	252 (1.33%)	180 (0.95%)	170 (0.90%)	81 (0.43%)	29 (0.15%)	19 (0.10%)	18,921		

Orange red indicates the tumor with the highest percentage.

### 3.2 Category analysis of malignant female reproductive system tumors across different age groups

Over the past 60 years, the Beijing Obstetrics and Gynecology Hospital has treated 94 patients aged 0–18 years with gynecological tumors. The most prevalent tumors in this age group were ovarian, trophoblastic, and vaginal. Among 2,596 patients aged 19–35 years, the leading types of tumors were cervical, ovarian, and trophoblastic. In the age brackets of 36–45 years, 46–55 years, 56–65 years, 66–75 years, and >76 years, there were 3,950, 6,009, 4,605, 1,468, and 199 patients, respectively. The distribution of various gynecological malignant tumors varied significantly according to the age group (*p* < 0.05). The three most common cancers were cervical, endometrial, and ovarian cancers. These tumors were most frequent in middle-aged women aged >36 years. Trophoblastic tumors predominantly affected young women of childbearing age between 19 and 35 years old, whereas other malignant tumors of the female reproductive system occurred at any age (Table 2).

**Table 2:** Category analysis of malignant tumors of the female reproductive system in different age groups, shows tumor distribution across age groups.

Age	Disease												
	Cervical cancer	Endometrial cancer	Ovarian cancer	Trophoblastic tumor	Vulvar cancer	Uterine sarcoma	Follopian tube cancer	Vaginal cancer	Residual vaginal cancer	Peritoneal cancer	Total	Chi-square value	*p*-value
0–18	2 (0.0%)	2 (0.0%)	74 (2.5%)	7 (1.0%)	0	0	1 (0.6%)	7 (8.6%)	0	1 (5.3%)	94 (0.49%)	3509.284	0.000
19–35	1,334 (13.1%)	162 (3.7%)	563 (19.3%)	495 (68.3%)	8 (3.2%)	19 (10.6%)	7 (4.1%)	6 (7.4%)	0	2 (10.6%)	2,596 (13.72%)		
36–45	2,578 (25.3%)	596 (13.7%)	571 (19.6%)	100 (13.8%)	28 (11.1%)	44 (24.4%)	16 (9.4%)	8 (9.9%)	7 (24.1%)	2 (10.5%)	3,950 (20.88%)		
46–55	3,082 (30.2%)	1,656 (38.1%)	928 (31.8%)	110 (15.2%)	69 (27.4%)	67 (37.2%)	66 (38.8%)	18 (22.2%)	9 (31.0%)	4 (21.1%)	6,009 (31.76%)		
56–65	2,313 (22.7%)	1,496 (34.4%)	575 (19.7%)	10 (1.4%)	83 (32.9%)	36 (20.0%)	54 (31.8%)	26 (32.1%)	7 (24.1%)	5 (26.3%)	4,605 (24.34%)		
66–75	789 (7.7%)	393 (9.0%)	183 (6.3%)	3 (0.4%)	43 (17.1%)	13 (7.2%)	24 (14.1%)	14 (17.3%)	2 (6.9%)	4 (21.1%)	1,468 (7.76%)		
≥76	96 (0.9%)	47 (1.1%)	25 (0.9%)	0	21 (8.3%)	1 (0.6%)	2 (1.2%)	2 (2.5%)	4 (13.8%)	1 (5.3%)	199 (1.05%)		
Total	10,194 (53.8%)	4,352 (23.0%)	2,919 (15.4%)	725 (3.8%)	252 (1.3%)	180 (1.0%)	170 (0.9%)	81 (0.4%)	29 (0.2%)	19 (0.1%)	18,921		

Orange red indicates the tumor with the highest percentage.

### 3.3 Average age of patients with malignant tumors of the female reproductive system at Beijing obstetrics and gynecology hospital in the last 60 years

**Figure 1:**
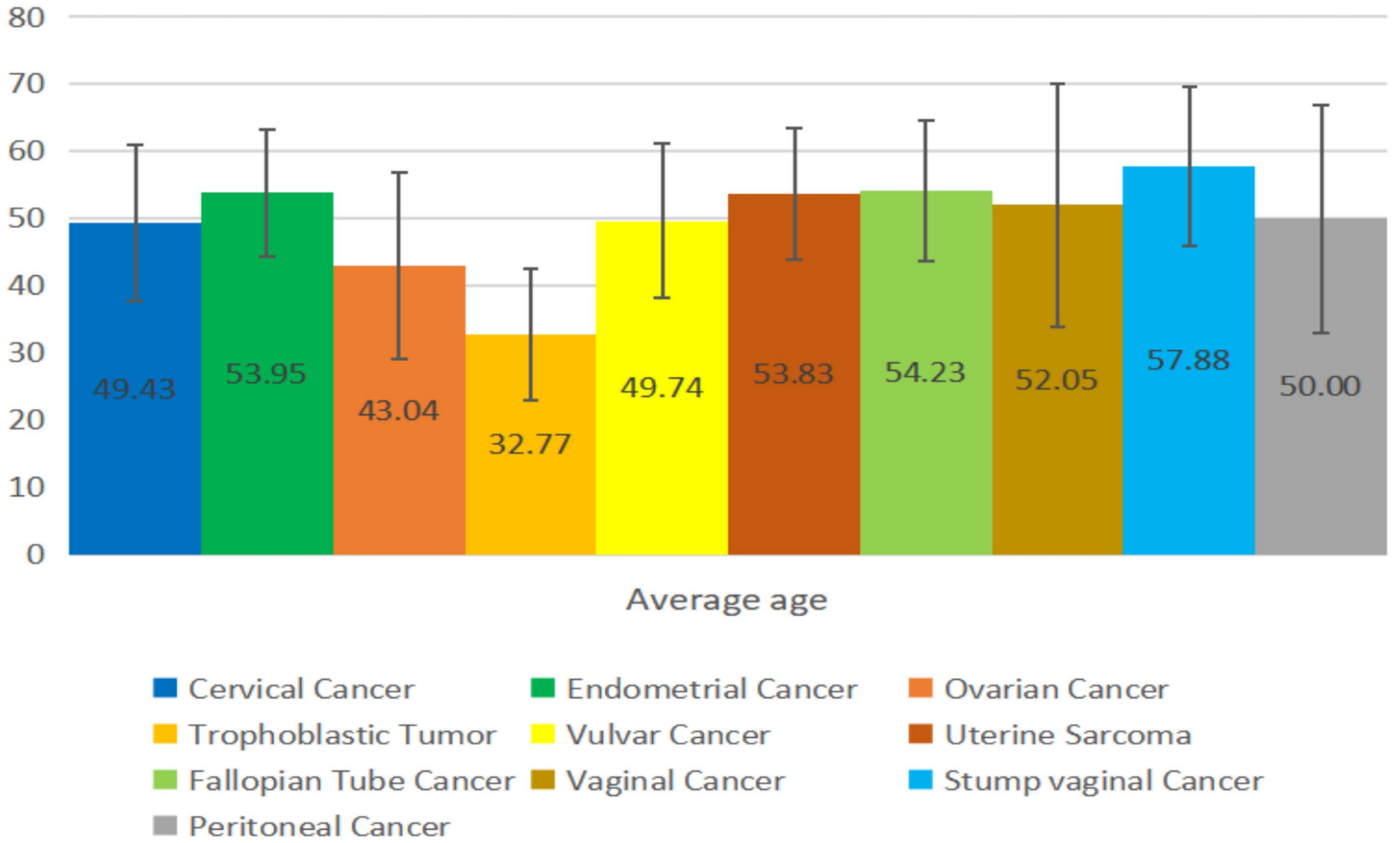
Average age of patients with malignant tumors of the female reproductive system.

The changes in the ages of patients with different tumor types at Beijing Obstetrics and Gynecology Hospital over the past 60 years showed that the average age was 49.43 ± 11.65 years for cervical cancer patients, 53.95 ± 9.45 years for endometrial cancer patients, 43.04 ± 13.79 years for ovarian cancer patients and 32.77 ± 9.76 years for trophoblastic cancer patients. The mean age of patients with vulvar cancer was 49.74 ± 11.44 years, that of patients with uterine sarcoma was 53.83 ± 9.78 years, that of patients with tubal cancer was 54.23 ± 10.51 years, that of patients with vaginal cancer was 52.05 ± 18.03 years, and that of patients with stump vaginal cancer was 57.88 ± 11.82 years. The average age of the patients with peritoneal carcinoma was 50.00 ± 17.00 years. The age at onset of trophoblastic tumors, the main malignant tumors in women of childbearing age, was relatively young. The age at onset of other tumors was relatively high, and most patients were perimenopausal (Figure 1).

### 3.4 Age changes in the three most common malignant tumors of the female reproductive system at Beijing obstetrics and gynecology hospital over the last 60 years

**Figure 2:**
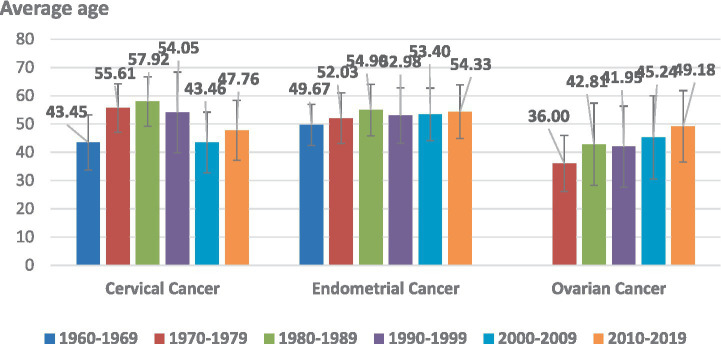
Age distribution changes associated with the three most common malignant tumors of the female reproductive system.

The three most common tumors in the Beijing Obstetrics and Gynecology Hospital over 60 years were cervical (53.88%, 10,194/18,921), endometrial (23.00%, 4,352/18,921), and ovarian (15.43%, 2,919/18,921) cancers. Over the past 20 years, there has been a trend toward younger age among patients with cervical cancer, whereas the average age of those diagnosed with endometrial and ovarian cancers have gradually increased (Figure 2).

### 3.5 Histopathological distribution of three major malignant tumors of the female reproductive system in the last 60 years

Over the past 60 years, Beijing Obstetrics and Gynecology Hospital has treated 10,194 patients with cervical cancer, 4,352 patients with endometrial cancer, and 2,919 patients with ovarian cancer. Squamous cell carcinoma (85.61%–100%) was the most prevalent cervical cancer subtype, followed by adenocarcinoma accounting for 0%–11.28%, adenosquamous carcinoma accounting for 0%–2.7%, mesenchymal tumor accounting for 0%–0.45%, and malignant melanoma accounting for 0%–0.32%. Adenocarcinoma (88%–100%) was the most common endometrial cancer subtype, followed by endometrial mesenchymal tumors (0%–10.39%), adenosquamous carcinoma (0%–3.72%), squamous cell carcinoma (0.1%–0.19%), and carcinosarcoma (0%–0.13%). Epithelial carcinoma was the main pathological ovarian cancer subtype, accounting for 74.77%–100%, followed by germ cell tumors accounting for 0%–17.5%, sex cord-stromal tumors accounting for 0%–12%, mesenchymal tumors accounting for 0%–1.97%, metastatic tumors accounting for 0%–0.96%, and melanoma accounting for 0%–0.07% (Table 3).

**Table 3:** Histopathological distribution of three major malignant tumors of the female reproductive system in the last 60 years.

Disease		Year						
		1960–1969	1970–1979	1980–1989	1990–1999	2000–2009	2010–2019	Total
Cervical cancer	Squamous cell carcinoma	67 (100.00%)	645 (97.00%)	1,523 (96.94%)	583 (89.83%)	1,663 (88.84%)	4,597 (85.61%)	9,078 (89.06%)
	Adenocarcinoma	0 (0%)	14 (2.10%)	42 (2.68%)	52 (8.02%)	156 (8.33%)	606 (11.28%)	870 (8.53%)
	Adenosquamous cell carcinoma	0 (0%)	2 (0.30%)	6 (0.38%)	13 (2.00%)	43 (2.30%)	145 (2.70%)	209 (2.05%)
	Mesenchymal tumor	0 (0%)	3 (0.45%)	0 (0%)	1 (0.15%)	4 (0.21%)	20 (0.37%)	28 (0.27%)
	Malignant melanoma	0 (0%)	1 (0.15%)	0 (0%)	0 (0%)	6 (0.32%)	2 (0.04%)	9 (0.09%)
	Total	67 (0.66%)	665 (6.52%)	1,571 (15.41%)	649 (6.37%)	1872 (18.36%)	5,370 (52.68%)	10,194 (100.00%)
Endometrial cancer	Adenocarcinoma	6 (100.00%)	68 (88.31%)	303 (91.54%)	504 (96.18%)	900 (90.09%)	2,309 (95.61%)	4,090 (93.98%)
	Mesenchymal tumor	0 (0%)	8 (10.39%)	14 (4.23%)	9 (1.72%)	54 (5.41%)	71 (2.94%)	156 (3.59%)
	Squamous cell carcinoma	0 (0%)	0 (0%)	1 (0.30%)	1 (0.19%)	1 (0.10%)	2 (0.08%)	5 (0.11%)
	Adenosquamous cell carcinoma	0 (0%)	0 (0%)	13 (3.93%)	9 (1.72%)	35 (3.50%)	10 (0.41%)	67 (1.54%)
	Carcinosarcoma	0 (0%)	1 (1.30%)	0 (0%)	1 (0.19%)	9 (0.90%)	23 (0.95%)	34 (0.78%)
	Total	6 (0.14%)	77 (1.77%)	331 (7.61%)	524 (12.04%)	999 (22.95%)	2,415 (55.49%)	4,352 (100.00%)
Ovarian cancer	Epithelial tumors	–	2 (100.00%)	188 (80.34%)	243 (74.77%)	701 (83.75%)	1,383 (90.93%)	2,517 (86.23%)
	Sex cord stromal tumor	–	0 (0%)	2 (0.85%)	39 (12.00%)	51 (6.09%)	25 (1.64%)	117 (4.01%)
	Malignant germ cell tumors	–	0 (0%)	42 (17.95%)	40 (12.31%)	70 (8.36%)	74 (4.87%)	226 (7.74%)
	Mesenchymal tumor	–	0 (0%)	2 (0.85%)	1 (0.31%)	7 (0.84%)	30 (1.97%)	40 (1.37%)
	Metastatic cancer	–	0 (0%)	0 (0%)	2 (0.62%)	8 (0.96%)	8 (0.53%)	18 (0.62%)
	Malignant melanoma	–	0 (0%)	0 (0%)	0 (0%)	0 (0%)	1 (0.07%)	1 (0.04%)
	Total	–	2 (0.07%)	234 (8.02%)	325 (11.13%)	837 (28.67%)	1,521 (52.11%)	2,919 (100.00%)

Orange red indicates the tumor with the highest percentage.

## 4 Discussion

The patients treated at our hospital reflect the characteristics of patients with malignant tumors of the female reproductive system in Beijing and throughout China. Since the establishment of the Department of Gynecological Oncology in 1970, the number of patients with malignant tumors of the female reproductive system has increased exponentially. The situation of patients with malignant tumors of the female reproductive system treated in our hospital over the past 60 years is significant for a preliminary understanding of the characteristics of female reproductive system tumors in Beijing and the future direction of disease control in Beijing. In addition, this study has several limitations. First, as a single-center study, our findings may not be generalizable to other regions. Second, referral bias may have overrepresented complex cases. Finally, diagnostic advancements over the past 60 years may have influenced case detection rates. Further multicenter studies are required to validate these findings.

### 4.1 Malignant female reproductive system tumor disease spectrum analysis from 1960 to 2019

The primary risk factors for cancer include genetic factors (4), environmental influences, and viral infections (5). The spectrum of cancer incidence in China is gradually moving toward that of developed countries such as the United States (6). Over the past 60 years, the number of diagnosed and treated malignant female reproductive system tumors has increased annually. With improvements in screening, diagnosis, and treatment, the number of patients has steadily increased. Over time, cervical cancer remained the most common female reproductive system tumor in China, while, endometrial and ovarian cancers, as genetically related tumors, steadily increased in incidence. Cervical, ovarian, and endometrial cancer incidences and mortalities were highest in areas with low human development indices. In areas with a high human development index, the incidence of cervical, endometrial, and ovarian cancers was high, while the mortality rate of cervical cancer was higher than that of ovarian and endometrial cancers, with slightly different distributions in different regions (7). In 2020, China’s human development index was 0.761, which is considered high. Data from our hospital show that, in terms of female reproductive system malignancies, the most common cancer in China is cervical cancer, followed by endometrial cancer, ovarian cancer, trophoblastic tumors, vulvar cancer, and uterine sarcoma. Cervical, ovarian, and endometrial cancer prevalences were representative of the international population in China. In summary, hereditary female reproductive system tumors (ovarian cancer and endometrial cancer) were relatively less affected by external factors such as the environment and policies and the increase in the prevalence of these tumors was in proportion to the population. Whereas Infectious tumor prevalence (cervical cancer) was closely related to policies, improved screening methods, sexual behavior, and living habits and had relatively large fluctuations in incidence.

### 4.2 Analysis of age and disease types associated with malignant tumors of the female reproductive system

The three most common tumors primarily occurred in middle-aged and elderly women (>36 years old), and the peak age for trophoblastic tumors was 19–35 years old. These findings aligned with the age distribution and trends of various tumors reported internationally (2). The American Cancer Society guidelines recommend that women aged 45–54 years undergo annual screening for reproductive system tumors. In the guidelines, women aged 40–44 years should be offered the option to begin annual screening, while women aged >55 years should either transition to biennial screening or be given the choice to continue with annual screening. Screening should persist as long as women are in good overall health and have a life expectancy of at least 10 years (8).

Trophoblastic neoplasia is primarily associated with pregnancy and childbearing, with the lowest average age at onset. Cervical, ovarian, and vulvar cancers typically develop during the perimenopausal period. Cervical and vulvar cancers are linked to human papilloma virus (HPV) infection, which develops slowly. Consequently, the peak age at onset of these cancers is approximately 45 years. The age at which females are most susceptible to different malignant reproductive system tumors was related to the distinct characteristics of each tumor.

### 4.3 Analysis of age changes in three major malignant tumors of the female reproductive system in the last 60 years

#### 4.3.1 Cervical cancer

Cervical cancer is the third leading cause of cancer-related death among women in low- and middle-income countries, accounting for 7.9% and 7.5%of the incidence and mortality, respectively, of women with cancer worldwide (9). Cervical cancer is rare in high-income countries due to improved reproductive health. Since low- and middle-income countries are undergoing economic transition, lifestyle risk factors, such as smoking, physical inactivity, being overweight, and poor reproductive health, are more prevalent leading to higher cervical cancer mortality (10). The major risk factor for cervical cancer is chronic infection with HPV (11), and 12 types of HPV have been classified by the International Agency for Research on Cancer as having definite carcinogenic effects on humans (12). HPV16 and 18 are responsible for 70% of cervical cancers worldwide (13). Approximately 291 million women, or 10.4% of the global population, are estimated to have cervical HPV infection (14). However, nearly 80–90% of infections are cleared by the body within a few years, and only women with persistent infection are at risk of cancer (15). Factors that increase the risk of cervical HPV infection include early sexual intercourse and having multiple sexual partners (16). The increase in the number of young women with cervical has been attributed to changes in sexual behavior and inadequate cervical screening, leading to an increased prevalence of high-risk HPV infection (17, 18).

Considering that liquid-based cytology became the primary screening method for cervical cancer in China in 2001, young women can now undergo comprehensive screening owing to routine examinations and high acceptance rates. Consequently, the patients diagnosed with cervical cancer are typically younger. The liberalization of the two-child policy in 2016 is likely to have resulted in increased sexual activity among young people. The HPV vaccine was also introduced in China in 2016 (19). Although the HPV vaccine can prevent 70–90% of cervical cancer cases, the vaccine does prevent all HPV infections that can cause cervical cancer. In addition, HPV vaccine coverage is not yet universal, likely leading to a small peak in cervical cancer incidence in China in the next 10 years and future trends toward diagnosis in younger patients and increasing prevalence of non-HPV infection-related cervical cancers. Both vaccination and screening will play vital roles in reducing the cervical cancer burden.

#### 4.3.2 Carcinoma of the corpus uteri (endometrial carcinoma)

Carcinoma of the corpus uteri accounts for approximately 4.8% and 2.1% of cancer incidence and mortality, respectively, in women worldwide (9). Most risk factors for carcinoma of the corpus uteri (the vast majority of which are endometrial carcinomas) are hormone-related and include being overweight, abdominal obesity, menopausal estrogen therapy, early menarche, late menopause, nonparitiy, polycystic ovary syndrome, and tamoxifen use (20). Excess weight accounts for 34% of all uterine cancers worldwide (21). Other risk factors include Lynch syndrome and diabetes (22). The factors associated with risk reduction include pregnancy, use of oral contraceptives, use of intrauterine devices, and physical activity (23–37). Trends in corpus uteri cancer are mainly driven by changes in the rates of obesity and hysterectomy, and reproductive/hormonal factors such as the stage of labor and use of menopausal hormone therapy (27). Since 2000, the incidences of uterine cancers have been increasing in the United States, Central and Eastern Europe, and several other European countries, likely due to the strong correlation between cancer and obesity, as well as rising obesity and declining birth rates in these regions (27, 28). In some historically low-risk regions, such as Asia, the incidence and patient age are also slowly increasing, and the mortality has increased with increasing incidence (28, 29). The aging population, rising number of obese individuals, and infertility are factors contributing to the increasing incidence of corpus carcinoma in China (21, 30). The trends in the age and annual incidence of endometrial cancer in our hospital are similar to those in China, possibly for the same reasons.

#### 4.3.3 Ovarian cancer

Ovarian cancer accounts for approximately 4.6% and 4.3% of morbidity and mortality, respectively, among women with cancer worldwide (8). A family history of breast or ovarian cancer is an important risk factor for ovarian cancer, with known genetic predispositions (primarily germline mutations in *BRCA1* or *BRCA2*) accounting for 10%–15% of cases (31). The incidence of ovarian cancer is increasing in women with Lynch syndrome (32). There are several hormone-related risk factors for ovarian cancer, such as menopausal hormone therapy (combination and estrogen only) and excess weight, as well as protective factors, such as pregnancy and oral contraceptives (20, 34, 35). It is estimated that the long-term use of oral contraceptives can reduce the risk of ovarian cancer by 33%. The risk reduction is greater for long-term oral contraceptive users (36, 37), and tubal ligation can also reduce the risk of ovarian cancer (33). Smoking is a risk factor of relatively rare mucinous neoplasms (38). The worldwide incidence and mortality from ovarian cancer have been declining over the past two decades due to the use of oral contraceptives and a decline in menopausal hormone therapy (39). The decline in mortality may also be attributed to improved treatment (40). Owing to the progress in screening and diagnostic methods and the increase in the proportion of elderly people in China, the number and age of patients with ovarian cancer in China have increased annually.

### 4.4 Changes in the pathological types of three major malignant reproductive system tumors in females from 1960 to 2019

According to the pathological classification of cervical cancer, squamous cell carcinoma was the most prevalent type. Endometrioid carcinoma primarily presented as an endometrial mesenchymal tumor, followed by squamous cell carcinoma. Epithelial ovarian cancer was the most common type of ovarian cancer, with germ cell tumors and sex cord-stromal tumors being the next most frequent. In the coming decade, the use of HPV vaccines and oral contraceptive pills, and implementation of primary prevention strategies for tumors could alter the pathological distribution of malignant female reproductive system tumors.

## 5 Prospects

In 2016, the HPV vaccine was introduced in China, and by 2019, the administration of China’s independently developed HPV vaccine was expected to significantly reduce the incidence of cervical cancer between 2040 and 2050, aligning with prevention and control efforts for cervical cancer in high-income countries. With a growing understanding of hereditary malignant tumors of the female reproductive system, along with the widespread adoption of genetic testing and increasing emphasis on the primary prevention of gynecological tumors, the incidence of endometrial and ovarian cancers is anticipated to gradually decline. Due to medical intervention and lifestyle modifications, the types and proportions of malignant female reproductive system tumors are expected to change in the future. The gradual evolution of the malignant female reproductive system tumor disease spectrum in China over the next decade will provide a foundation for adjusting the prevention and control policies for these tumors.

## Data Availability

The raw data supporting the conclusions of this article will be made available by the authors, without undue reservation.
